# Evaluation of Antinociceptive Effects of Chitosan-Coated Liposomes Entrapping the Selective Kappa Opioid Receptor Agonist U50,488 in Mice

**DOI:** 10.3390/medicina57020138

**Published:** 2021-02-04

**Authors:** Liliana Mititelu Tartau, Maria Bogdan, Beatrice Rozalina Buca, Ana Maria Pauna, Cosmin Gabriel Tartau, Lorena Anda Dijmarescu, Eliza Gratiela Popa

**Affiliations:** 1 Department of Pharmacology, Faculty of Medicine, “Grigore T. Popa” University of Medicine and Pharmacy, 700115 Iasi, Romania; liliana.tartau@umfiasi.ro (L.M.T.); beatrice-rozalina.buca@umfiasi.ro (B.R.B.); ana-maria-raluca-d-pauna@umfiasi.ro (A.M.P.); cosmin-gabriel.ig.tartau@students.umfiasi.ro (C.G.T.); 2 Department of Pharmacology, Faculty of Pharmacy, University of Medicine and Pharmacy, 200349 Craiova, Romania; 3 Department of Obstetrics-Gynecology, Faculty of Medicine, University of Medicine and Pharmacy, 200349 Craiova, Romania; lorenadijmarescu@yahoo.com; 4 Department of Pharmaceutical Technology, Faculty of Pharmacy, “Grigore T. Popa” University of Medicine and Pharmacy, 700115 Iasi, Romania; eliza.popa@umfiasi.ro

**Keywords:** selective kappa opioid receptor agonist, U50,488, chitosan-coated liposomes, tail flick test, writhing test, antinociceptive

## Abstract

*Background and Objectives*: The selective kappa opioid receptor agonist U50,488 was reported to have analgesic, cough suppressant, diuretic and other beneficial properties. The aim of our study was to analyze the effects of some original chitosan-coated liposomes entrapping U50,488 in somatic and visceral nociceptive sensitivity in mice. *Materials and Methods*: The influence on the somatic pain was assessed using a tail flick test by counting the tail reactivity to thermal noxious stimulation. The nociceptive visceral estimation was performed using the writhing test in order to evaluate the behavioral manifestations occurring as a reaction to the chemical noxious peritoneal irritation with 0.6% acetic acid (10 mL/kbw). The animals were treated orally, at the same time, with a single dose of: distilled water 0.1 mL/10 gbw; 50 mg/kbw U50,488; 50 mg/kbw U50,488 entrapped in chitosan-coated liposomes, according to the group they were randomly assigned. *Results*: The use of chitosan-coated liposomesas carriers for U50,488 induced antinociceptive effects that began to manifest after 2 h, andwere prolonged but with a lower intensity than those caused by the free selective kappa opioid in both tests. *Conclusion*: In this experimental model, the oral administration of nanovesicles containing the selective kappa opioid agonist U50,488 determined a prolonged analgesic outcome in the tail flick test, as well as in the writhing test.

## 1. Introduction

Nanomedicine offers particularly valuable research and practical application tools in clinical medicine, improving the currently used methods of prevention, diagnosis, targeted therapy in various pathological conditions, from simple inflammatory illnesses to neoplastic diseases [1].

The application of nanotechnology in the development of new drug formulations has opened a new area of researching the prolonged release of different pharmacologically active substances.

The drug-carrying nanoformulations consist of solid biodegradable particles, with the size varying between 10 and 1000 nm, in which the active ingredient is dissolved, trapped, or encapsulated and/or in which the active substance is absorbed or attached [2]. The most important technological advantages of nanoparticles used as drug carriers are represented by the possibility of entrapping of both hydrophilic and hydrophobic agents, high stability of the obtained systems, easy administration by various routes, and increased transport capacity in the body [3].

Generally, the nanoparticulate systems are used to achieve an extended release of active substances, to prolong drug effect in the targeted tissue, to reduce its adverse effects [1].

Liposomes are systems with a high variation in shape and surface electric charge, but also in the composition of the lipid component, which determines the differences in permeability through biological membranes. The composition of the lipid bilayer has an essential role in modulating the fluidity of the vesicular membrane; this element contributesto the stability of liposomes, andalso to the kinetic model of releasing the encapsulated components [4,5].Vesicles can carry both hydrophilic and hydrophobic molecules. In the preparation of vesicles, a very important role is played by the geometry of amphiphilic molecules, because only cylindrical molecules form bilayers. These can be both lipids (two-tailed biological molecules) and a mixture of single-tailed surfactants of opposite charge [3,4].

Kappa opioid receptors (KORs) are expressed in brain areas which express analgesia and affect cognition, but also in other areas throughout the body [6,7]. Like mu opioid receptors, activation of KORs determines antinociceptive effects [6,8]. The highest efficacy in visceral pain models is recorded in KOR agonists from all opioid analgesics [9].

The trans-3,4-dichloro-N-methyl-N-[2-(1-pyrrolidinyl)cyclohexyl]-benzeneacetamide derivative U-50,488 is a selective KOR agonist, possessing analgesic, cough suppressant, and diuretic properties. It also suppresses the psychostimulant reward through the inhibition of dopamine signaling in the addiction pathways [10,11,12], with obvious influence in decreasing amphetamine-induced psychomotor disturbances [13], in preventing cocaine [14] or nicotine-induced motor troubles [15], and in reducing the charges of morphine [16] or fentanyl [17] self-administration in rodents. Other research has evidenced the beneficial effects of U50,488 on L-dopa attenuation of motor dyskinesia in rodents and primates with experimental-induced Parkinsonian manifestations [18,19].

The purpose of our study was to evaluate the effects of some original chitosan-coated liposomes entrapping U50,488 in somatic and visceral nociceptive sensitivity in mice.

## 2. Materials and Methods

### 2.1. Design and Characterization of U50,488 Entrapped in Chitosan-Coated Liposomes

The lipid used, L-α-phosphatidylcholine (L-α-lecithin), chitosan, chloroform and U50,488 were purchased from Sigma Chemical Co., Steinheim, Germany. We used chitosan with N-de-acetylation degree of 28%, apolydispersity index of 3.26, with average molecular mass Mn = 94,810, and average gravimetric mass Mw = 309,900. Chitosan was dissolved in 10% (w/w) acetic acid. To obtain chitosan solution with a 1% (w/w) homogeneity, the liquid was stirred at room temperature over 24 h and the excess of gas was removed by centrifugation at 1500 rpm for 30 min.

Initially, the lipid (L-α-lecithin) was dissolved in chloroform; subsequently, a dry lipid film was achieved upon evaporation of the solution. A solution of U50,488 in distilled water was added for the film hydration, and continuous stirring at low rotation induced the formation of multilamellar vesicles. The lipid solution was sonicated, in order to convert the multilamellar vesicles to unilamellar vesicles [20]. Then, a colloid solution (with a ratio of 3:2 lipid vesicles:chitosan (v/v) was prepared by adding 0.5% chitosan [21,22]. In order to remove the acidity and to achieve a neutral pH, the soft matter vesicles entrapping U50,488 were dialyzed for 4 h, by using dialysis tubing semi-permeable membranes, 12,000 Da MWCO (type D6191-25EA, Sigma Chemical Co., Steinheim, Germany). 

The size distribution and Zeta potential were determined using Zetasizer Malvern Nano ZS Zen-3500 Apparatus (Worchestershire, UK). The transmission spectra were recorded using a Hewlett Packard 8453 UV–VIS spectrophotometer (Waldbronn, Germany), to highlight the encapsulation of substances in vesicles and to establish the elimination curves. With a Nikon Ti Eclipse optical microscope (Tokyo, Japan), the obtained nanoparticles were visualized directly, the images being taken with a Coolpix 950 digital camera.

### 2.2. Nociceptive Sensitivity Testing

#### 2.2.1. Animals

For this study, white Swiss mice (weighting 20–25 g, evenly distributed by gender) were used. The animals were housed in standard laboratory conditions and received standard laboratory chow and water ad libitum. The study was approved (Protocol No. 19157/19.10.2009) by the Ethics Committee for Research of the University of Medicine and Pharmacy “Grigore T. Popa” from Iasi, Romania, in compliance with the regulations of the International Association for the Study of Pain regarding the handling of laboratory animals [23,24].

#### 2.2.2. The Tail Flick Test

A total of 21 animals, assigned in 3 groups of 7 white Swiss mice (with 4 male and 3 female each), were used. At the same time, the animals were treated orally (using an eso-gastric device) with a single dose as follows:

Group 1 (DW): distilled water 0.1 mL/10 gbw;

Group 2 (U50,488): 50 mg/kbw U50,488;

Group 3 (U50,488-ves): 50 mg/kbw U50,488 entrapped in chitosan-coated liposomes.

The influence on the somatic pain was assessed using tail flick test by counting the tail reactivity to thermal noxious stimulation (54 °C). The latency period of the response was measured before the experiment (baseline), at 15, 30, 60, 90 min, 2, 4, 6, 8, 10, 12 h after the administration of the substances. The recommended cut-off time of 12 s was used to prevent tissue damages. In this animal model, a prolongation of the response latency expressedthe analgesic effect, while a decrease in the reactivity time suggested the hyperalgesic effects of the tested substances [25,26].

To quantify the intensity of antinociception, the latency of the response was converted to percentage maximum possible effect (%MPE), according to the formula [27]:

*%MPE = [(measured latency − baseline latency) × 100]: [cut-off time − baseline latency]*

The results obtained in tail flick test were presented as mean ± standard deviation (SD) of the mean latencies of the response, respective to the mean percentage maximum possible effect.

#### 2.2.3. The Writhing Test

For the experiment, 210 animals divided in 10 lots were used. Each lot consisted of identical 3 groups of 7 white Swiss mice (20–25 g): Group 1 (DW): distilled water 0.1 mL/10 gbw; Group 2 (U50,488): 50 mg/kbw U50,488; Group 3 (U50,488-ves): 50 mg/kbw U50,488 entrapped in liposomes.

The nociceptive visceral estimation was performed using the writhing test in order to evaluate the behavioral manifestations occurring as a reaction to the chemical noxious peritoneal irritation with 0.6% acetic acid (10 mL/kbw) [28].

The tested substances were orally given at the same time at the beginning of the experiment. Acetic acid was intraperitoneally injected 5 min prior to each moment of the determination, according to the following scheme: lot I—half an hour; lot II—1 h; lot III—2 h; lot IV—3 h; lot V—4 h; lot VI—5 h; lot VII—6 h; lot VIII—8 h; lot IX—10 h; lot X—12 h after the administration of substances. The animals were placed in glass cages, and the number of writhes were counted for 5 min.

In this experimental model, the diminution in the number of writhes was indicative of analgesia, while the increasein the behavioral manifestations number correspondedto a hyperalgesic effect of the tested substances.

In addition, the antinociceptive effects of U50,488 free or entrapped in nanoparticles were assessed by calculating the pain inhibition percentage (PIP) [29]:

*PIP = (No. control writhes − No. counted writhes for each treated group)/No. counted writhes for each treated group) × 100*

The results collected in the writhing test were expressed as mean ± the standard deviations (SD) of the mean number of writhes, respective to thepercentage values of pain inhibition.

### 2.3. Statistical Analysis

The data were centralized and statistically processed using the ANOVA (analysis of variance) method and the Bonferroni test with the SPSS version 22.0. The Bonferroni correction, as a post-hoc test, was performed, in order to diminish the error rate when analyzing multiple data points. Values of the coefficient *p* (probability) below 0.05, were considered statistically significant, compared to the control group.

## 3. Results

### 3.1. Characteristics of Chitosan Vesicles

**Figure 1:**
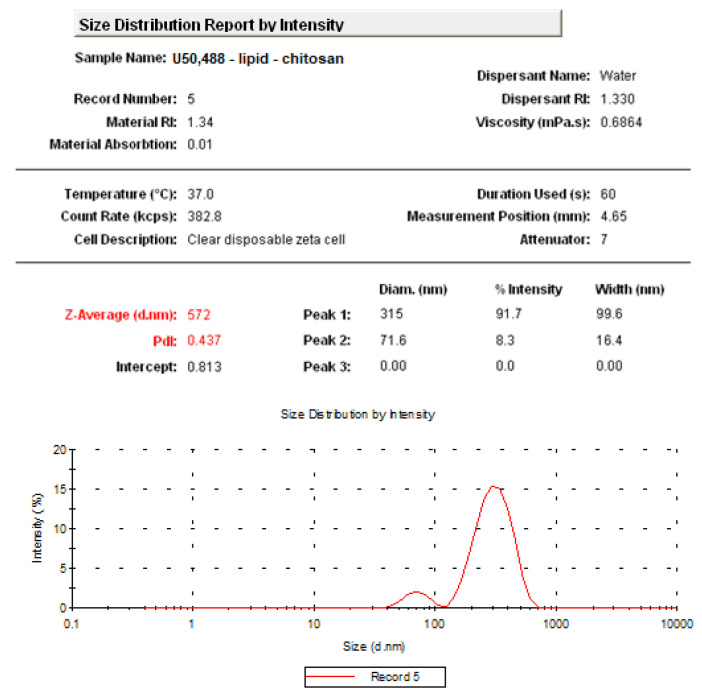
Size distribution by number of U50,488 vesicles in aqueous solution.

The obtained chitosan-coated liposomes entrapping U50,488 proved to have a mean size of 572 nm (Figure 1). The use of chitosan was associated by an augmentation of the vesicles dimension, determining a stiffening of the bilayers.

**Figure 2:**
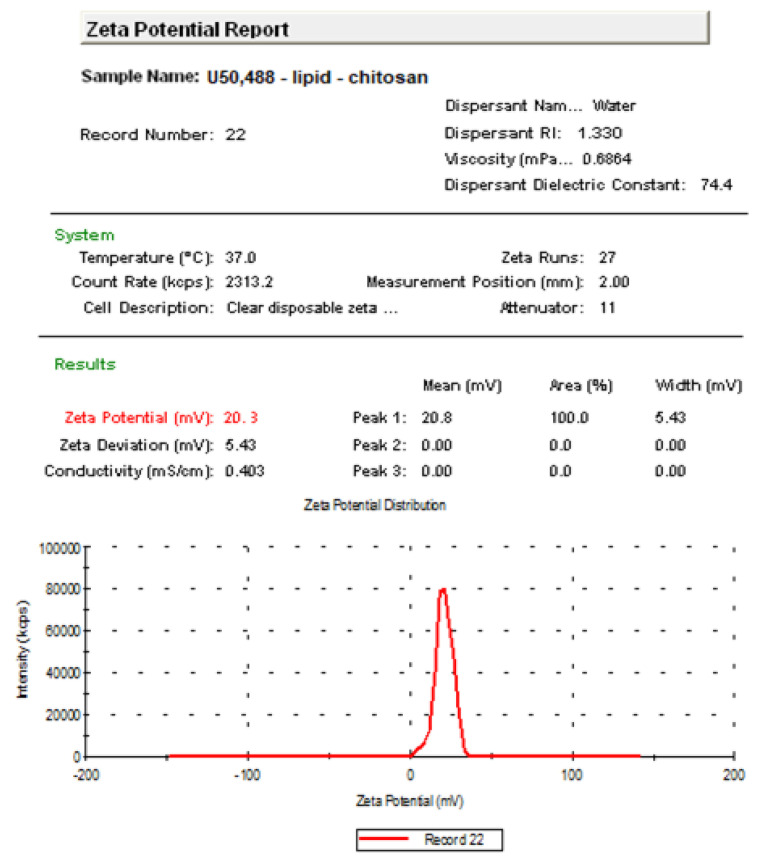
Distribution of the U50,488 vesicle’s Zeta potential.

The measured mean Zeta potential of the vesicles containing U50,488 was +20.3 mV (Figure 2).

### 3.2. The Tail Flick Test

**Figure 3:**
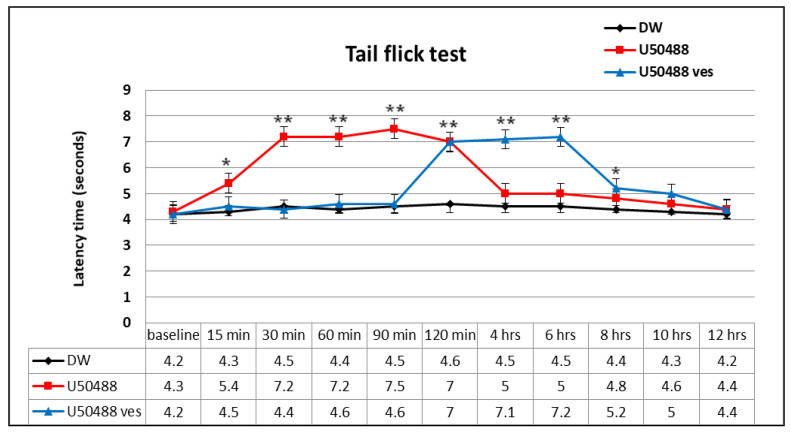
The effects of U50,488 entrapped in chitosan-coated liposomes in the tail flick test. Each point represents the mean ± SD of the latency time (seconds) for seven mice in a group. * *p* < 0.05, ** *p* < 0.01 compared to the control.

**Figure 4:**
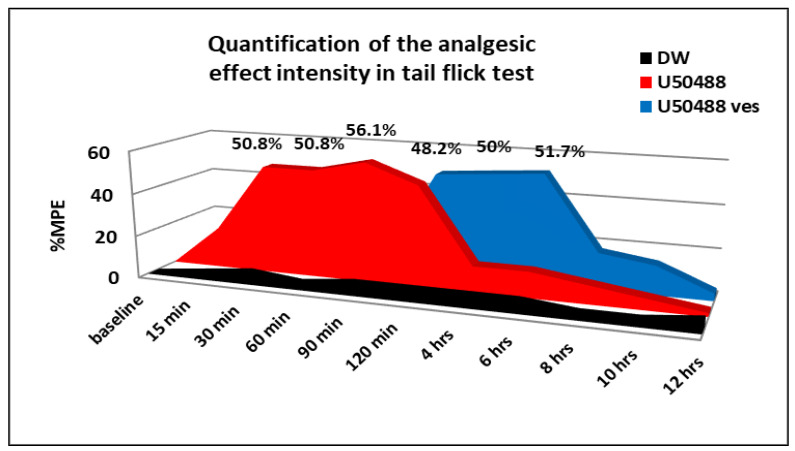
The percentage maximum possible effect (%MPE) of U50,488 entrapped in chitosan-coated liposomes in the tail flick test. Each point represents the mean ± SD of the percentage maximum possible analgesic effect for seven mice in a group.

The administration of U50,488 caused a rapid and statistically significant increase (** *p* < 0.01) in the response latency, an effect lasting approximately 120 min after the substance administration (Figure 3). By performing Bonferroni’s test, statistically significant variations were revealed between U50,488 group and distilled water group at 15 min (* *p* < 0.05), 30 min (** *p* < 0.01), 60 min (** *p* < 0.01), 90 min (** *p* < 0.01) and 120 min (** *p* < 0.01) in the experiment. Its maximum possible analgesic effect was achieved at 90 min (%MPE = 56.1 ± 11.3%) (Figure 4).

The use of U50,488 chitosan-coated liposomes induced a prolongation in the latency period of time, which began at 2 h and lasted for about 8 h in this experimental cutaneous pain model in mice (Figure 3). By applying Bonferroni’s multiple comparison, statistically significant differences were evidenced between group treated with U50,488 vesicles and the group receiving distilled water, 120 min (** *p* < 0.01), 4 h (** *p* < 0.01), 6 h (** *p* < 0.01) and 8 h (* *p* < 0.05) after administration ofthe substance. The manifested maximum possible antinociceptive effect of U50,488 entrapped in chitosan-coated liposomes was observed after 6 h in the experiment (%MPE = 51.7 ± 10.5%) (Figure 4).

### 3.3. The Writhing Test

**Figure 5:**
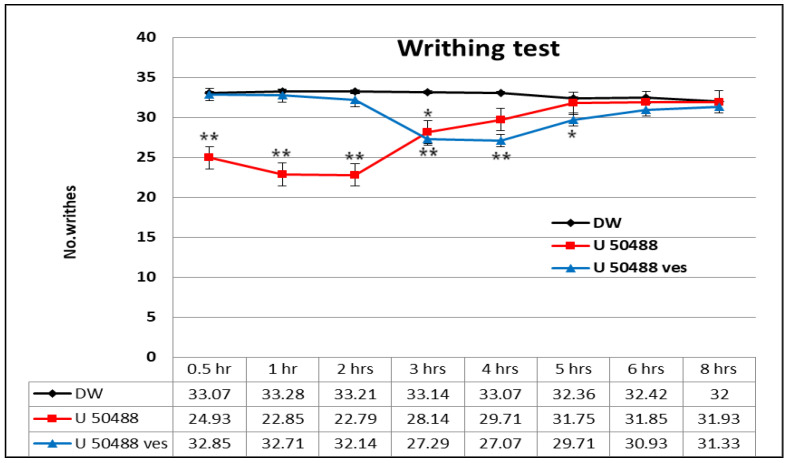
The effects of U50,488 entrapped in chitosan-coated liposomes in the writhing test. Each point represents the mean ± SD of the number of writhes for seven mice in a group. * *p* < 0.05, ** *p* < 0.01 compared to the control.

**Figure 6:**
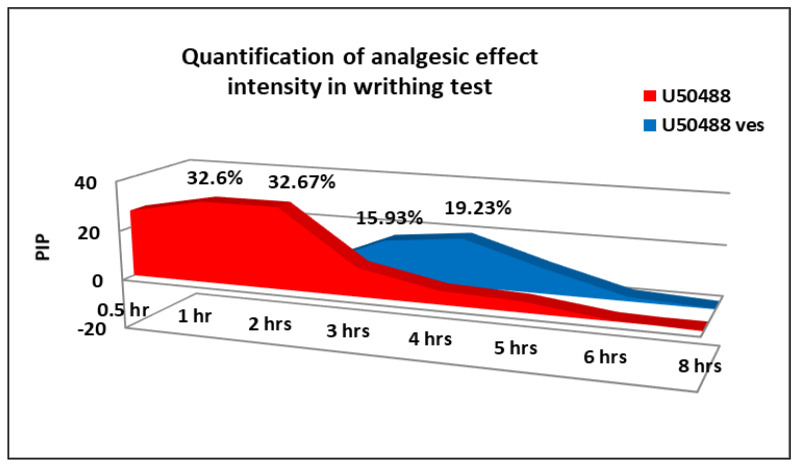
The pain inhibition percentage (PIP) of U50,488 entrapped in chitosan-coated liposomes in writhing test. Each point represents the mean ± SD of the maximum pain inhibition percentage for seven mice in a group.

U50,488 produced a rapid and gradual decrease in the number of writhes, statistically significant compared to the control group (** *p* < 0.01) in the first 2 h and prolonged at 3 h (* *p* < 0.05) in the writhing test (Figure 5). The multiple data processing showed statistically significant variations between U50,488 mice and distilled water-treated mice at 0.5 h (** *p* < 0.01), 1 h (** *p* < 0.01), 2 h (** *p* < 0.01) and 3 h (* *p* < 0.05), respectively, by the Bonferroni test. The maximum pain inhibition percentage was found after 2 h in the experiment (32.67 ± 7.5%) (Figure 6).

The treatment with U50,488 entrapped in chitosan-coated liposomes was correlated with a substantial reduction in the behavioral manifestations number compared to the control (* *p* < 0.05) within 3–5 h in this visceral pain model in mice (Figure 5). By using the Bonferroni post-test, statistically significant variances were noted between U50,488 vesicle group and distilled water group:3 h (** *p* < 0.01), 4 h (** *p* < 0.01), and 5 h (* *p* < 0.05) after the application of the chemical visceral stimulus. The maximum pain inhibition percentage was observed at 4 h in the experiment (19.23 ± 3.7%) (Figure 6).

## 4. Discussion

Our previous research evidenced that the agent U50,488 was effectively incorporated in the inner aqueous layer of the vesicles. Physicochemical analysis also revealed that the dimensions of U50,488 vesicles varied from tens to hundreds of nanometers, with an average size of 572 nm. The addition of chitosan in lipid-U50,488 vesicle solution was associated with an increase in the nanoparticle dimensions and a stiffening of the bilayers. The vesicles had a mean Zeta potential of +20.3 mV, thus proving an incipient stability of the colloidal solution [30].

Corroborating the transmission spectra of the agent released from the vesicles with the calibration curve of free kappa opioid, a high efficiency of trapping U50,488 in the chitosan-coated liposomes was shown. The analysis of the release curve from the vesicles showed that in the first 3 h, approximately 50% of the U50,488 incorporated amount was released, and after 7–8 h, 80% of the total amount of substance was found in the release medium [30].

The anterior results, in concordance with the literature-communicated data, have shown significant analgesic effects of selective kappa opioid agents, exhibited in various experimental acute and chronic pain models in laboratory animals, and also in clinical trials [31,32]. U50,488 was found to reduce pain transmission in different pain models in rodents, such as the hot plate test [33], tail flick test [34,35], paw pressure test [36,37], tail immersion test [38], formalin test [39,40], and cyclophosphamide-induced cystitis in mice [35].

In vitro studies, as well as in vivo experiments, have suggested that various ionic channels are involved in the mediation of both somatic and visceral pain. Su X. et al. demonstrated that U50,488 substantially blocks the voltage-activated sodium current in colon sensory neurons in vitro and reduces the in vivo visceral behavioral manifestations due to mechanical noxious colorectal distention in rats [41].

In vitro studies performed on isolated rat dorsal root ganglia neurons transfected with complementary deoxyribonucleic acid for enhanced green fluorescent protein proved antinociceptive activity, which was mediated by the inhibition of calcium channels and modulation of the calcium currents in sensory neurons involved in the nociceptive transmission [40,41,42].

So far, there are no communications regarding the design of nanoparticles entrapping the selective kappa opioid agonist U50,488, although the effects of this compound have been studied over time.

We imagined an original method of incorporating the selective kappa opioid agonist U50,488 into vesicles that will transport and prolong drug release in the targeted area, depending on environmental conditions (pH, ionic strength, temperature). The protocol for obtaining these nanoparticulate systems involves the use of association polymers based on the unmodified, biocompatible, and biodegradable polymer chitosan. 

Chitosan is a deacetylated product of chitin (an abundant natural resource) insoluble in water and most organic solvents. Its derivatives have been developed as nanomaterials, such as nanoparticles, microspheres, hydrogels, and micelles [43,44].

Due to its low toxicity, biodegradability and biocompatibility, chitosan has been increasingly studied in a wide range of pharmaceutical, biomedical, and biotechnological fields, including gene therapy, bone regeneration, food industry, and agriculture [45,46]. Chitosan displays hemostatic and hastening wound healing effects, anti-hypercholesterolemic effects, bioactivity, chemotactic actions, antimicrobial activity, immunostimulation, enzymatic biodegradability, mucoadhesion, andepithelial permeability [47,48,49,50].

For obtaining chitosan-based nanoparticles, several methods have been described: polyelectrolyte complexation, emulsion-droplet coalescence, emulsion crosslinking, ionotropic gelation, reverse micellization, and precipitation [48,51].

Because they have showed strong evidence for crossing the blood–brainbarrier [52], chitosan nanoparticles are studied for therapeutic compounds transportation as a potential therapy in Parkinson’s disease, Alzheimer’s disease, schizophrenia, gliomas, and cerebral ischemia [53].

The association of chitosan to the designed vesicles produced stiffening of the bilayer and stabilization of the particles, making them spherical and more confined; the obtained systems corresponding to the criteria of the colloidal solutions. 

In the experimental somatic pain model, the opioid kappa agonist U50,488 produced a significant and rapid antinociceptive effect, even after 15 min, which was manifested for 2 h. After this period, the effect was obviously reduced, reaching levels comparable to those found in the control group. The highest intensity of analgesic action (56.1 ± 11.3%) was observed at 90 min in the experiment. In the tail flick test, the administration of U50,488 incorporated into chitosan-coated liposomes was associated with a significant antinociceptive effect, which began after 120 min and persisted for up to 8 h, being the most intense (with a maximum analgesic effect of 51.7 ± 10.5%) at 6 h in the experiment. After this time, the effects of U50,488 vesicles were similar to those highlighted in U50,488 and the control.

In the experimental model of visceral pain, the administration of U50,488 caused a rapid antinociceptive effect, which persisted for 3 h. The strongest analgesic action of this opioid kappa agonist expressed after 2 h in the experiment, when a pain inhibition percentage of 32.67 ± 7.5% was highlighted. After 3 h, the effects of U50,488 were similar to those of the group that received distilled water in the writhing test. The use of U50,488 vesicles determined a significant antinociceptive effect, which manifested 3 h after the administration of the chitosan-coated liposomes and lasted for 5 h. The maximum intensity of analgesic action was found after 4 h in the experiment, when a maximum percentage of pain inhibition of 19.23 ± 3.7% was recorded. After 5 h, the effects of U50,488 incorporated into chitosan-coated liposomes were comparable to those of the free substance and to that of the control over the entire time interval up to 12 h.

The oral administration of liposomes containing the selective kappa opioid agonist U50,488 was accompanied by prolonged analgesic effects inthe tail flick test, as well as in the writhing test; however, a latency period of occurrence of these effects was noted. In both cutaneous and visceral experimental pain models, the delayed onset of the antinociceptive effects, as well as the prolongation of the analgesic activity of the liposomes entrapping U50,488, can be attributed to the particularities of the release of the active substance from the chitosan-coated liposomes.

The reference therapy for medium to severe pain is represented by opioid analgesics; thus, the opioid receptors are of utmost importance in modern medicine [54]. Pain, both acute and chronic, is associated with low productivity and high costs in healthcare services [55], which together with the classical opioid therapy’s limitations has led to intensified efforts for the discovery of analgesics with improved profile [56]. Over the last ten years, KOR agonists have been studied as alternatives to mu opioid receptors analgesics, due to their minimal respiratory and gastrointestinal adverse effects and their low abuse potential [57,58].

## 5. Conclusions

Original nanoparticles capable of incorporating U50,488 with high efficiency were developed. The use of chitosan-coated liposomes as carriers for U50,488 induced antinociceptive effects that began to manifest after 2 h, were prolonged, but had alower intensity than those caused by the free selective kappa opioid in the tail flick test, as well as in the writhing test.

We can conclude that, in our experimental conditions, the administration of U50,488 entrapped in chitosan-coated liposomes presented the advantage of prolonged drug release, comparedwith non-entrapped substance in both somatic and visceral pain models in mice.

## Data Availability

Not applicable.
